# Effects of the *Agrobacterium rhizogenes rolC* Gene Insertion on Secondary Metabolites Profile and In Vitro Biological Activity of *Acmella oleracea* (L.) R.K. Jansen

**DOI:** 10.3390/plants14091373

**Published:** 2025-05-01

**Authors:** Priscilla Paola Bettini, Martina Imbesi, Patrizia Bogani, Valentina Maggini, Filippo Firenzuoli, Fabio Firenzuoli, Domenico Trombetta, Antonella Smeriglio

**Affiliations:** 1 Department of Biology, University of Florence, Via Madonna del Piano 6, Sesto F.no, 50019 Florence, Italy; p.bettini@unifi.it (P.P.B.); patrizia.bogani@unifi.it (P.B.); valentina.maggini@unifi.it (V.M.); 2 Department of Chemical, Biological, Pharmaceutical and Environmental Sciences, University of Messina, Viale Ferdinando Stagno d’Alcontres 31, 98166 Messina, Italy; martina.imbesi@studenti.unime.it; 3 CERFIT, Research and Innovation Center in Phytotherapy and Integrated Medicine, Careggi University Hospital, Largo Giovanni Alessandro Brambilla 3, 50134 Florence, Italy; filippo.firenzuoli@unifi.it (F.F.); fabio.firenzuoli@unifi.it (F.F.); 4 Department of Health Sciences (DSS), Section of Anesthesiology, Intensive Care and Pain Medicine, Largo Giovanni Alessandro Brambilla 3, 50134 Florence, Italy

**Keywords:** *Acmella oleracea* (L.) R.K. Jansen, aerial parts, root, in vitro plant culture, *Agrobacterium rhizogenes*, *rolC* gene, polyphenols, alkylamides, antioxidant activity, anti−inflammatory activity

## Abstract

This study investigates the transformation of *Acmella oleracea* with the *Agrobacterium rhizogenes rolC* gene and evaluates its impact on phytochemical composition and biological activity. A total of 480 plant nodes were subjected to *Agrobacterium*−mediated transformation, leading to the regeneration of 35 putative transgenic plants. Molecular analysis confirmed the presence of the *rolC* transgene in 17 clones, of which four (C123, C127, C129, and C132) exhibited *rolC* mRNA expression. Phytochemical profiling of hydroalcoholic extracts of aerial parts (AP) and roots (R) revealed significant differences (*p* ≤ 0.05) between transgenic and non-transgenic plants (CTR). Compared to non−transgenic plants, transgenic AP exhibited lower total phenolic content but retained or increased flavonoid concentrations, particularly flavan−3−ols, whereas R extracts consistently showed reduced secondary metabolite levels. LC−DAD−ESI−MS analysis identified a diverse metabolite profile, with AP being notably rich in flavonoids (48.65%) and alkylamides (32.43%), including spilanthol. Functional assessments across antioxidant and anti−inflammatory assays demonstrated that R extracts exhibited stronger bioactivity compared to AP extracts, as indicated by lower IC_50_ values (0.004–2.18 mg/mL for R vs. 0.007–7.24 mg/mL for AP). However, iron−chelating capacity was higher in AP extracts, correlating with flavonoid concentration. Hierarchical clustering confirmed that transgenic lines C123 and C127 most closely resembled the control, while C129 and C132 displayed distinct metabolic profiles. These findings highlight *rolC*’s role in modulating secondary metabolite synthesis, influencing both the phytochemical composition and functional properties of *A. oleracea* extracts.

## 1. Introduction

*Acmella oleracea* (L.) R.K. Jansen, belonging to the Asteraceae family, is an herbaceous plant characterized by small yellow inflorescences and creeping growth habit [1,2,3,4,5,6,7,8,9]. It is traditionally used in folk medicine and as an edible species due to its bioactive compounds, notably spilanthol [6,7,9,10,11]. First identified by Gerber in 1903 [12], spilanthol is primarily found in the flowers, leaves, and stems [3,6,7,11,13,14,15] though it has also been detected in the roots [16,17]. Moreover, its accumulation in in vitro cell cultures has been extensively documented [2,3,6,11].

Due to its pharmacological significance—exhibiting a range of effects typical of alkylamides [18]—various protocols have been developed to optimize the production and extraction of spilanthol. In addition to recent in vivo treatments using biostimulants [19], in vitro plant cultures represent an effective method for generating, within a short period, large numbers of selected plants under standardized growth conditions [2,7,20,21,22,23,24]. In vitro techniques can be coupled to metabolic engineering with the aim of introducing new biosynthetic pathways or known modulators of secondary metabolism into medicinal plants, improving their nutritional composition and health benefits [25,26]. In this frame, transformation with *Agrobacterium rhizogenes* has been widely employed to enhance secondary metabolite production in medicinal plants [25,27,28].

*A. rhizogenes rol* genes (*rolA*, *B*, *C*, and *D*) have a pleiotropic effect on plant morphology and physiology, and their effect on secondary metabolites, in particular for *rolB* and *rolC*, is well documented [29].

Several studies have investigated the production of bioactive metabolites from *A. oleracea* using different in vitro culture systems or plant organs. Hairy root cultures have been explored for their ability to produce alkylamides such as spilanthol, showing promising results in terms of yield and stability [2]. Additionally, elicitation strategies—including treatments with methyl jasmonate and salicylic acid—have been applied to cell and tissue cultures to stimulate secondary metabolite biosynthesis, leading to enhanced antioxidant and anti−inflammatory activities [6,14]. Recent studies have further emphasized the potential of *A. oleracea* as a source of bioactive compounds through sustainable biotechnological approaches, including supercritical CO_2_ extraction and biostimulant treatments aimed at boosting alkylamide content [10,11]. Despite these advances, no previous research has applied genetic transformation with the *A. rhizogenes rolC* gene in *A. oleracea* to modulate the secondary metabolism. Thus, our study represents the first attempt to enhance the production of bioactive compounds in this species through a *rolC*−based, food−grade biotechnological approach.

Indeed, while the *rolC* gene has been widely used in other plant taxa, such as *Artemisia annua* and *Artemisia carvifolia* to enhance the production of the antimalarial drug artemisinin [10,30], as well as in *Lactuca sativa* to improve antioxidant and anti−inflammatory properties [31], its application to *A. oleracea* had not been reported prior to this study. Given the high pharmacological relevance of alkylamides, such as spilanthol, and the increasing interest in food−grade bioactive extracts, our work aims to fill this gap. Specifically, we focused on modulating the biosynthesis of secondary metabolites and evaluating the resulting changes in phytochemical composition and biological activities (antioxidant and anti−inflammatory) of food−grade extracts (ethanol:water, 80:20 *v*/*v*) obtained from both aerial parts and roots. The use of food−safe solvents and extraction processes compliant with safety standards, ensures that the resulting extracts are suitable for applications in nutraceutical, food, and cosmetic industries, promoting sustainable biotechnological approaches for the development of health−promoting herbal products.

## 2. Results

### 2.1. Transformation and Molecular Analysis of rolC−Transgenic Plants

**Figure 1:**
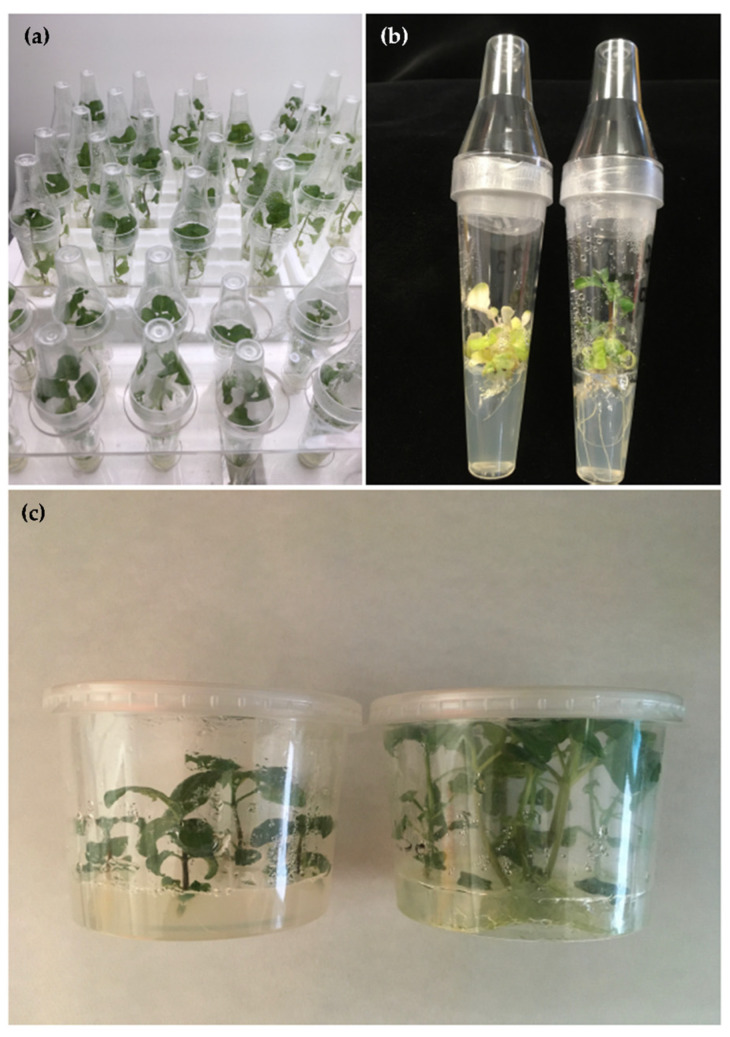
Transformation of *A. oleracea*. (**a**) Seed−derived plantlets; (**b**) Regenerated shoots after transformation on selective medium; (**c**) Transgenic clone rolC127 (left) and untransformed regenerated control (right).

Of the 480 nodes subjected to *Agrobacterium* transformation, 35 regenerated shoots were obtained (transformation efficiency: 7.3%), compared to 11 regenerated shoots from the untransformed controls (55% regeneration rate) (Figure 1b,c).

**Figure 2:**
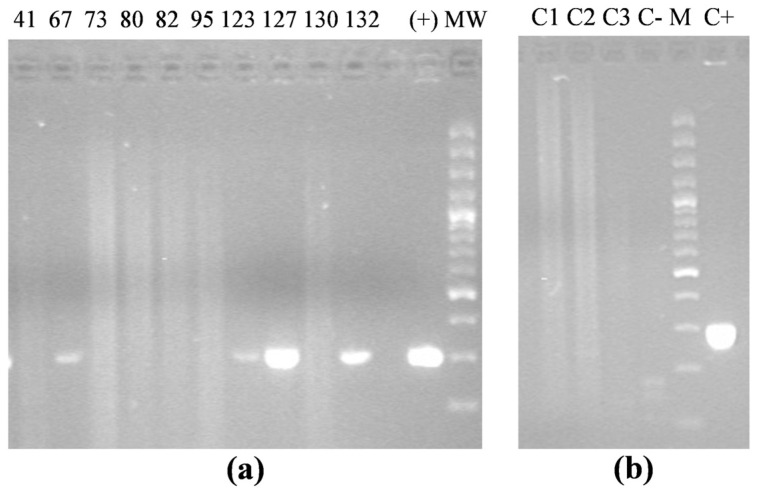
Direct PCR on leaf tissue from transformed (**a**) and control (**b**) *A. oleracea* plants with primers for *A. rhizogenes rolC* gene. +—positive control (*rolC* gene cloned in pUC18 plasmid); MW—molecular weight marker (GeneRuler 100 bp Plus DNA ladder, Thermo Scientific).

PCR analysis confirmed the integration of the *rolC* gene in 17 putative transgenic plants, while no amplification was detected in control samples (Figure 2).

Among these, four clones (C123, C127, C129, and C132) showed detectable *rolC* mRNA expression levels by quantitative real−time PCR, with clone C127 exhibiting the highest expression (9.14 fg/μg RNA), followed by C123 (1.6 fg/μg), C132 (0.282 fg/μg), and C129 (0.105 fg/μg) (Table 1).

### 2.2. Extraction and Phytochemical Characterization of Food−Grade Extracts

The ultrasound-assisted extraction of aerial parts and roots yielded high extraction efficiencies, ranging from 31.84% to 35.67% for aerial parts and 44.07% to 54.51% for roots across the different lines.

In aerial part extracts, total phenolic content ranged from 2.62 to 4.48 g gallic acid equivalent (GAE)/100 g dry extract (DE). Extracts from clones C123 and C132 had phenolic levels comparable to the control (4.33 ± 0.13 and 4.48 ± 0.26 vs. 4.22 ± 0.34 g GAE/100 g DE), while C127 and C129 exhibited significant reductions (*p* < 0.001).

Total flavonoid content was significantly higher in C123 and C127 compared to the control: 2.97 ± 0.22 and 2.46 ± 0.02 vs. 2.01 ± 0.14 g Rustin equivalent (RE)/100 g DE; *p* < 0.01, highlighting a selective enhancement of this subclass of polyphenols (Table 2). Similarly, flavan−3−ol content, assessed through the vanillin index, increased significantly in C123 and C127 compared to the control: 2.66 ± 0.03 and 1.84 ± 0.02 vs. 1.61 ± 0.03 g catechism equivalent (CE)/100 g DE; *p* < 0.001 and *p* < 0.05, respectively) (Table 2). For root extracts, all transgenic clones showed a marked reduction in total phenols and flavonoids compared to the control (*p* < 0.001), with C123 roots recording 4.27 ± 0.18 g GAE/100 g DE and 0.85 ± 0.01 g RE/100 g DE, respectively (Table 2).

The LC−DAD−ESI−MS analysis (Table 3 and Table 4) confirmed a richer metabolite diversity in aerial part extracts compared to roots, with 37 and 16 identified compounds, respectively. In aerial extracts, flavonoids accounted for 43% of identified metabolites, followed by alkylamides (35%) and phenolic acids (16%). Notably, quercetin derivatives predominated among flavonoids, while spilanthol among alkylamides (Table 3). In root extracts, alkylamides were more prominent (44%), with lower overall phenolic diversity compared to aerial parts. Spilanthol was detected but at much lower concentrations (Table 4).

Quantitative analysis revealed that aerial part extracts contained significantly higher spilanthol levels than roots (Table 5). In the aerial parts, spilanthol content was highest in the control (5.70 ± 0.08 g/100 g extract), followed by C132 (4.88 ± 0.05 g/100 g), while C129 showed a marked reduction (0.17 ± 0.01 g/100 g; *p* < 0.001). Roots exhibited considerably lower spilanthol amounts, ranging from 0.019 to 0.077 g/100 g (Table 5).

**Figure 3:**
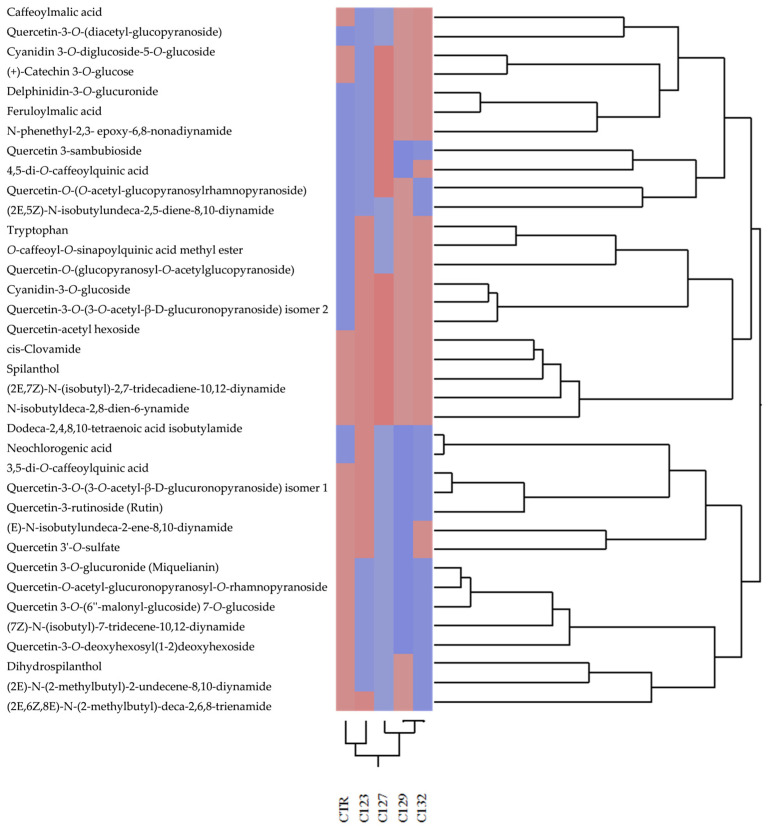
Hierarchical clustering analysis of the phytochemical profile detected in hydroalcoholic extracts of the aerial parts of transgenic (C123, C127, C129, and C132) and non−transgenic (CTR) *A. oleracea* plants. Different colors represent the degree of similarity among samples, with similar colors indicating closer phytochemical profiles and distinct colors highlighting greater differences.

**Figure 4:**
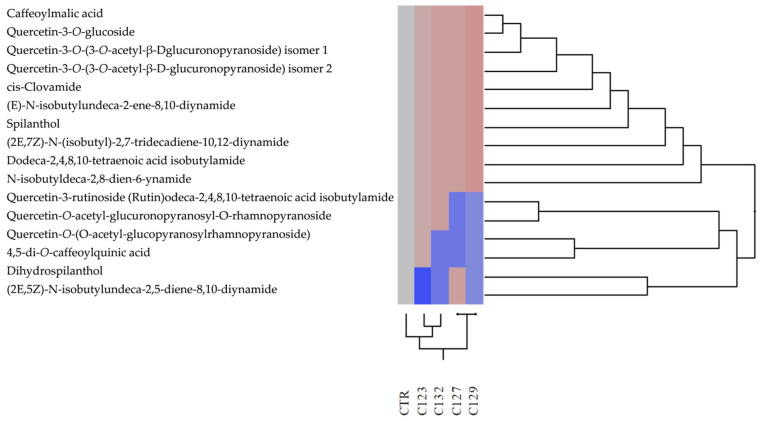
Hierarchical clustering analysis of the phytochemical profiles detected in hydroalcoholic root extracts of transgenic (C123, C127, C129, and C132) and non−transgenic (CTR) *A. oleracea* plants. Different colors represent the degree of similarity among samples, with similar colors indicating closer phytochemical profiles and distinct colors highlighting greater differences.

Hierarchical clustering based on the phytochemical profile positioned C123 as the closest to the control for both aerial parts and roots, followed by C127. In contrast, C129 and C132 formed a distinct cluster, highlighting significant metabolic differentiation (Figure 3 and Figure 4).

### 2.3. Evaluation of Antioxidant and Anti-Inflammatory Activity

**Figure 5:**
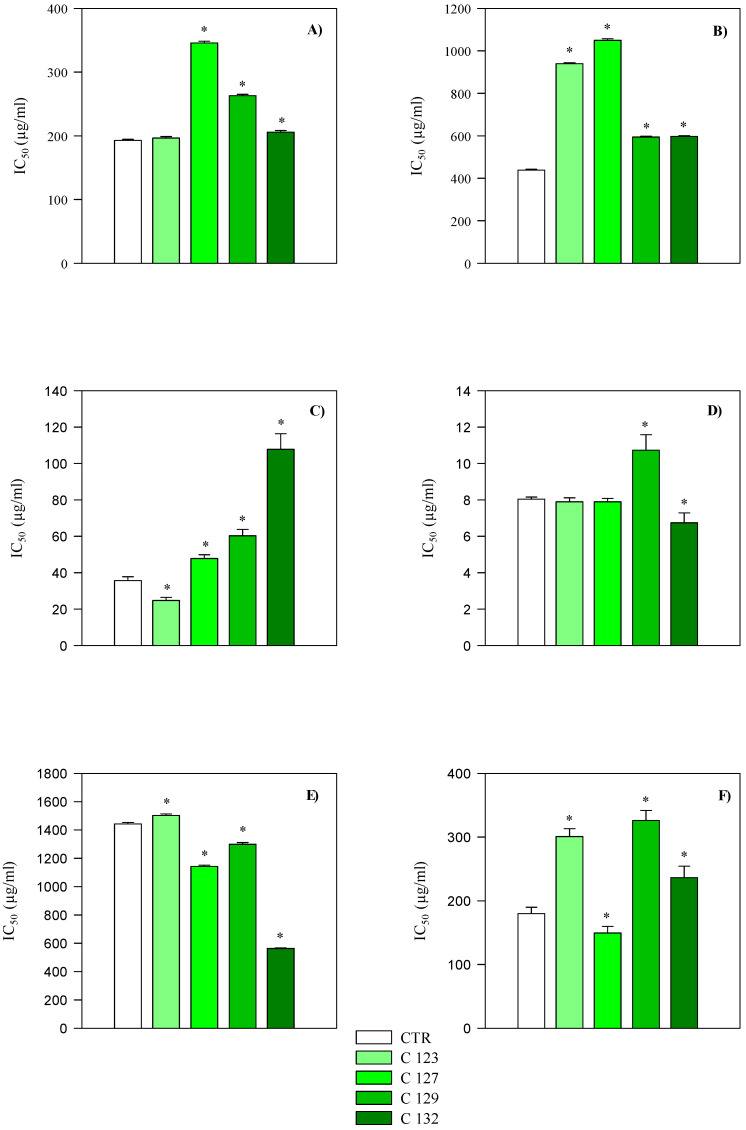
Comparison of antioxidant activity of hydroalcoholic extracts of aerial parts of transgenic (C123, C127, C129 and C132) and non−transgenic (CTR) *A. oleracea* plants. Results are expressed as IC_50_ and represent the mean ± standard deviation of three independent experiments in triplicate (*n* = 3). (**A**) FRAP; (**B**) DPPH; (**C**) TEAC; (**D**) ORAC; (**E**) β-Carotene bleaching; (**F**) Iron-chelating activity. * *p* < 0.001 vs. CTR.

**Figure 6:**
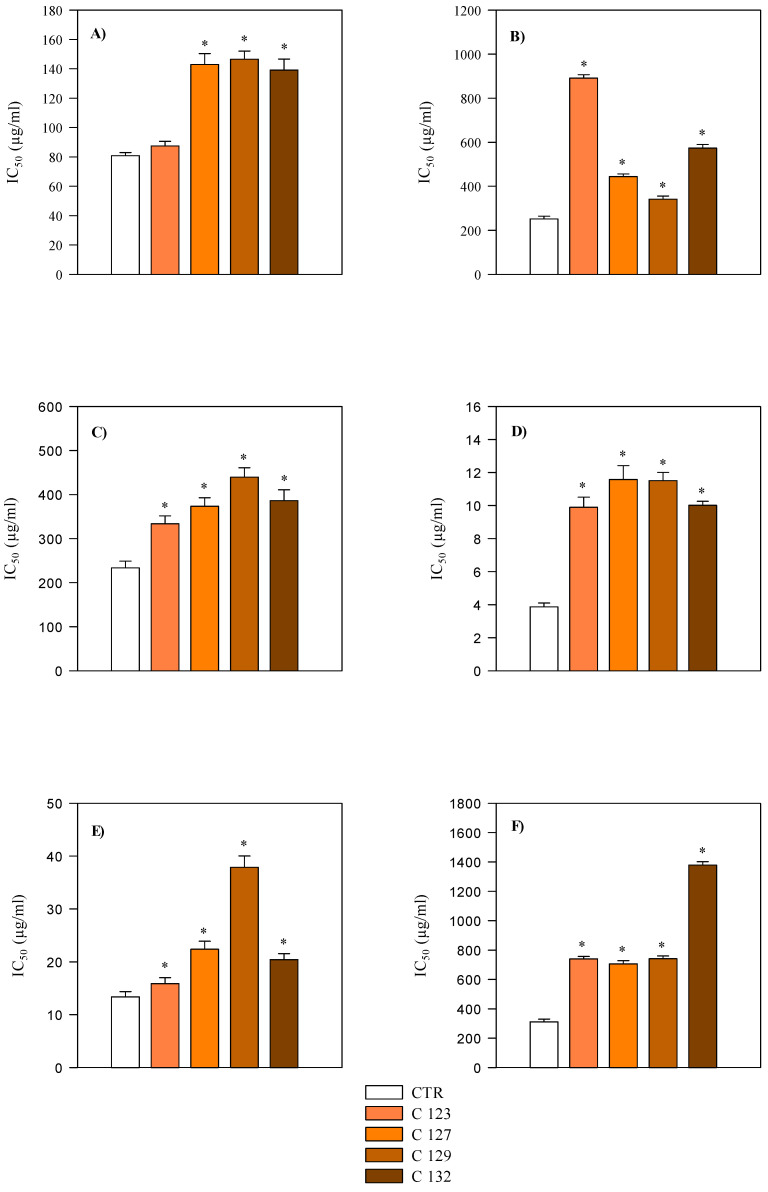
Comparison of antioxidant activity of hydroalcoholic extracts of transgenic (C123, C127, C129, and C132) and non−transgenic (CTR) *A. oleracea* roots. Results are expressed as IC_50_ and represent the mean ± standard deviation of three independent experiments in triplicate (*n* = 3). (**A**) FRAP; (**B**) DPPH; (**C**) TEAC; (**D**) ORAC; (**E**) β-Carotene bleaching; (**F**) Iron-chelating activity. * *p* < 0.001 vs. CTR.

For each assay, statistical analysis was conducted separately for aerial part and root extracts by comparing each transgenic clone to the corresponding non−transgenic control. In the aerial part extracts, antioxidant assays (FRAP, DPPH, TEAC, ORAC, BCB, and ICA) showed variable responses among the clones. Extracts from C123 and C127 generally retained antioxidant capacity similar to the control in DPPH, FRAP, and TEAC assays, while C129 and C132 exhibited significantly higher half-inhibitory concentration (IC_50_) values, indicating reduced antioxidant potency (Table 6, Figure 5). In root extracts, the antioxidant activity was consistently stronger compared to aerial parts, with lower IC_50_ values observed in FRAP, TEAC, and ICA assays (*p* < 0.001) (Table 6, Figure 6). However, iron−chelating activity was higher in aerial parts, in line with their greater flavonoid content (Table 6).

Overall, C123 root extracts displayed the most comparable antioxidant activity to the control, while C129 and C132 roots showed significantly different profiles (Figure 6).

**Figure 7:**
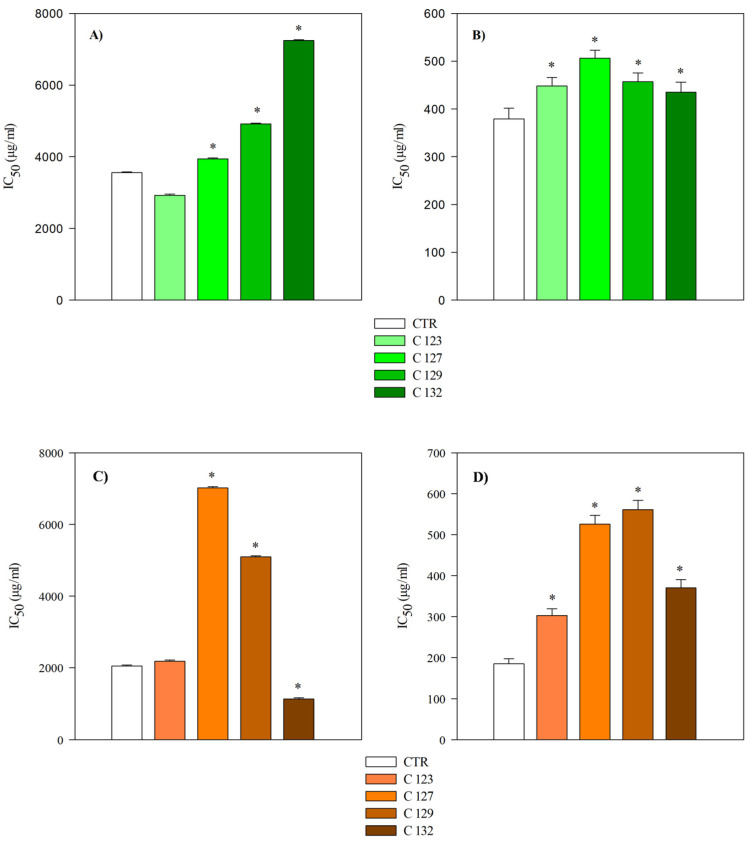
Comparison of the anti−inflammatory activity of hydroalcoholic extracts of aerial parts (**A**,**B**) and roots (**C**,**D**) of transgenic (C123, C127, C129, and C132) and non−transgenic (CTR) *A. oleracea* plants. Results are expressed as IC_50_ and represent the mean ± standard deviation of three independent experiments in triplicate (*n* = 3). Heat-induced BSA denaturation assay (**A**,**C**); Protease inhibition assay (**B**,**D**). * *p* < 0.001 vs. CTR.

Anti−inflammatory assays (ADA and PIA) revealed that root extracts were significantly more potent than aerial parts in inhibiting BSA denaturation (*p* < 0.001) (Table 7, Figure 7). Among aerial part extracts, C123 demonstrated anti−inflammatory activity comparable to the control, whereas C132 had a significantly higher IC_50_ value, indicating lower efficacy. Root extracts from C127 and C129 displayed the most pronounced anti−inflammatory effects, with IC_50_ values of 0.72 and 0.60 mg/mL, respectively, substantially lower than the control (2.05 mg/mL). These results support that *rolC* transformation selectively enhanced biological activities in certain transgenic lines, particularly at the root level.

## 3. Discussion

This study aimed to evaluate the effects of *A. rhizogenes rolC* gene insertion on the phytochemical profile and biological activities of *A. oleracea* extracts derived from aerial parts and roots. The findings clearly demonstrate that the transformation impacted the two plant parts differently, both in terms of secondary metabolite composition and functional properties.

In line with our findings, previous studies have demonstrated that hydroalcoholic extracts obtained using food−grade ethanol−water mixtures effectively preserve the phytochemical integrity of medicinal plants, ensuring their suitability for applications in the food and nutraceutical sectors. For instance, Dias et al. [14] successfully extracted spilanthol and other alkylamides from *A. oleracea* using supercritical CO_2_ and hydroalcoholic solvents, emphasizing the importance of non−toxic, food−safe extraction systems for preserving bioactivity. Similarly, Barbosa et al. [18] highlighted that ethanol-based extracts of *A. oleracea* maintained significant antioxidant and anti−inflammatory activity, supporting their potential use in functional foods and cosmetic formulations.

Additionally, Uthpala and Navaratne [9] reviewed the use of *A. oleracea* as an emerging food source, reinforcing that extracts obtained through food−grade processes are crucial for developing safe, edible, and therapeutic products. These studies, employing methodologies comparable to ours, validate the approach taken in the present research and strengthen the claim that the extracts produced herein possess the necessary food−grade quality for safe human consumption and further development into health−promoting products.

Phytochemical analysis revealed distinct metabolic profiles between aerial parts and roots. Aerial part extracts exhibited a higher number and diversity of secondary metabolites, with flavonoids accounting for 43% of the identified compounds, predominantly quercetin derivatives. Spilanthol, the characteristic alkylamide of *A. oleracea*, was also abundantly present in aerial parts, particularly in the control and C132 extracts.

In contrast, root extracts displayed a reduced diversity of metabolites (16 identified compounds), with alkylamides representing the most abundant class (44%). Despite the lower total phenolic content, root extracts maintained a significant presence of quercetin derivatives and spilanthol, although at considerably lower concentrations compared to aerial parts. These results suggest that the aerial parts retain a more complex and flavonoid-rich phytochemical profile, while the roots prioritize alkylamide accumulation, likely as a result of tissue-specific metabolic regulation.

Antioxidant activities observed reflect the phytochemical differences between plant parts. Aerial part extracts exhibited moderate antioxidant capacity in DPPH, FRAP, and TEAC assays, correlating with their higher flavonoid and phenolic acid content. Particularly, iron-chelating activity was significantly stronger in aerial part extracts, consistent with the presence of catechol−containing flavonoids such as quercetin derivatives, known for their potent metal chelation ability.

Root extracts, despite their lower phenolic content, demonstrated superior antioxidant capacity in assays based on electron or hydrogen atom transfer mechanisms (DPPH, TEAC, ORAC, and BCB). These findings suggest that alkylamides and possibly other root−specific compounds may contribute significantly to radical scavenging activities and highlight the complexity of the antioxidant mechanisms beyond polyphenol concentration alone. Significant differences between transgenic clones and the non-transgenic control were mainly observed in root extracts, where clones such as C127 and C129 displayed markedly enhanced antioxidant and anti-inflammatory activities (*p* < 0.001), while in aerial parts the differences were less consistent and generally smaller. In addition, when comparing aerial part and root extracts within the same clones, root extracts consistently exhibited lower IC_50_ values, suggesting a stronger overall bioactivity at the root level, independently of the genetic transformation. 

A similar pattern was observed in the anti−inflammatory assays. The aerial part extracts exhibited moderate inhibition of protein denaturation and protease activity, with some variability among clones. C123 maintained anti-inflammatory properties similar to the control, whereas C132 showed a reduction in efficacy. Root extracts, however, demonstrated significantly higher anti-inflammatory activity across all tests. Particularly, clones C127 and C129 exhibited stronger inhibition of heat−induced BSA denaturation compared to the control. This enhanced activity might be attributed to the synergistic effects of residual flavonoids and alkylamides, or to other root−specific bioactive compounds.

In agreement with the phytochemical profiles identified by LC−DAD−ESI−MS analysis, the differences in biological activities between transgenic and non-transgenic lines can be correlated with specific variations in key metabolite concentrations. Aerial part extracts from transgenic lines such as C123 and C127 exhibited selective enrichment in quercetin derivatives, including rutin and quercetin glucosides, despite an overall decrease in total phenolic content. These compounds, known for their strong antioxidant and metal−chelating properties [31,32], likely contributed to the retained antioxidant capacity observed in certain assays (e.g., FRAP and TEAC), despite reduced phenolic diversity. Moreover, the levels of spilanthol, a major alkylamide with documented anti−inflammatory activity [6], were significantly reduced in some transgenic lines (notably C129), which may partially account for the decreased efficacy observed in anti−inflammatory assays such as ADA and PIA. Conversely, root extracts, although characterized by lower overall phenolic diversity, maintained a relatively higher proportion of alkylamides, including spilanthol and its derivatives, which could explain their superior anti−inflammatory and radical−scavenging activities compared to aerial part extracts. The observed increase in specific flavonoids and phenolic acids in transgenic lines, particularly in root extracts, is consistent with the known role of *rolC* in modulating secondary metabolite pathways, including the upregulation of flavonoid biosynthesis [33,34].

The variability in IC_50_ values across different assays and extracts, reflects both biological and methodological factors. Biologically, the intrinsic differences between aerial parts and roots, combined with the varying degrees of *rolC* gene expression among transgenic lines, led to substantial heterogeneity in the content and distribution of key bioactive compounds, particularly flavonoids and alkylamides [34,35]. Methodologically, the antioxidant and anti−inflammatory assays employed in this study rely on distinct mechanisms of action—such as electron transfer, hydrogen atom transfer, or protein stabilization—that are differentially influenced by the specific chemical profiles of the extracts [36,37]. Consequently, the broad range of IC_50_ values accurately reflects the complex interplay between metabolite diversity and assay sensitivity, further underscoring the need for a comprehensive multi-assay evaluation approach when assessing the biological potential of plant−derived extracts [38]. The enhanced antioxidant and anti−inflammatory activities observed in certain transgenic clones, especially in root extracts, may thus be attributed, at least in part, to the *rolC*−induced alteration of the phenolic profile, favoring compounds with strong bioactive properties [34]. These results confirm that *rolC* transformation can differentially modulate the biological activities of distinct plant tissues, affecting their potential therapeutic applications.

Overall, the food−grade extracts from aerial parts and roots of *A. oleracea* exhibited complementary phytochemical and biological profiles. Aerial part extracts, characterized by high flavonoid and spilanthol content and strong iron−chelating capacity, are promising candidates for the development of antioxidant nutraceuticals and functional foods.

Root extracts, with their superior antioxidant and anti−inflammatory activities, suggest potential applications in formulations aimed at managing oxidative stress and inflammation−related conditions.

These findings highlight the importance of selecting specific plant parts according to the desired functional properties and support the relevance of food−grade extraction methodologies in preserving bioactive compound integrity for safe human use.

Overall, our results demonstrate that *rolC*−mediated genetic transformation can enhance the biosynthetic potential of *A. oleracea* in vitro, leading to modified secondary metabolite profiles and improved biological activities. These findings represent a promising step towards the biotechnological development of plant−derived products with potential applications in health−related fields, although further studies on whole plants and field conditions, as well as on specific molecular mechanisms underlying these observations, remain to be elucidated and are necessary to validate these outcomes.

## 4. Materials and Methods

### 4.1. Plant Transformation

**Figure 8:**
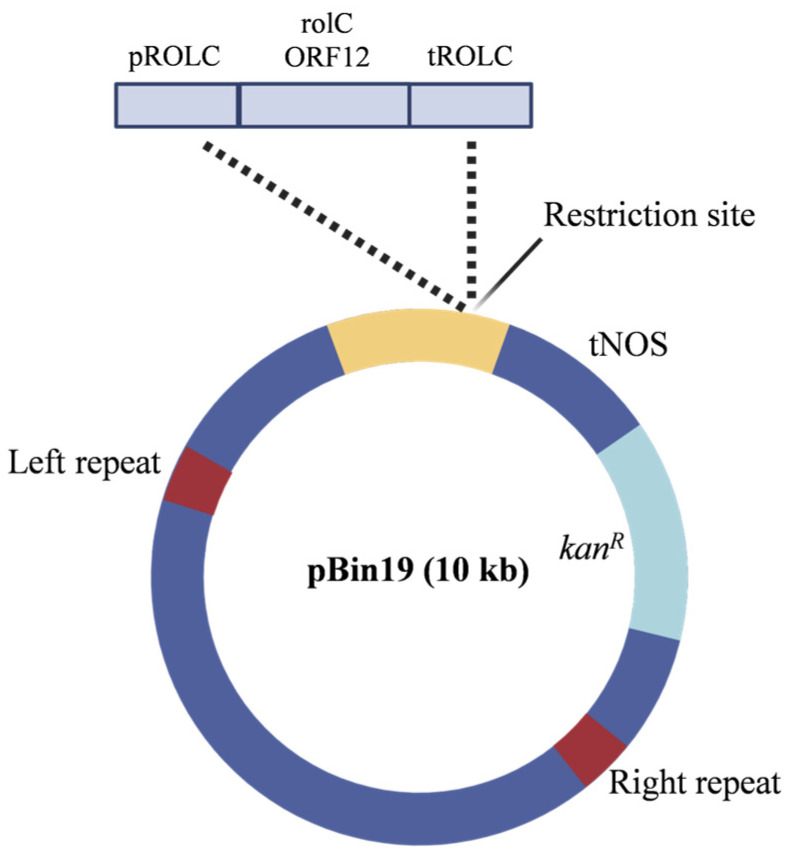
Binary vector Bin19 containing the *Hind*III-*Eco*RI fragment (1860 bp) from *A. rhizogenes* 1855, corresponding to the natural promoter (pROLC), the coding sequence (*rolC—*ORF12*)* and the terminator region (tROLC) for the *rolC* gene conjugated into *A. tumefaciens* LBA4404 strain. *kan^R^*—kanamycin resistance gene; pNOS—nopaline synthase gene promoter; tNOS—nopaline synthase gene terminator.

*A. oleracea* seeds (Saflax-Frank Laue, Münster, Germany) were surface−sterilized as described [39], sown onto Petri dishes containing LS medium (Duchefa Biochemie B.V., Haarlem, The Netherlands) with the addition of 30 g/L sucrose and 0.8% Phyto Agar (Duchefa Biochemie B.V.), and incubated in a growth chamber at 24 ± 1 °C. Germinated seedlings were transferred to De Wit culture tubes (Lab Associates B.V., Oudenbosch, The Netherlands) on the same medium (Figure 1a). After 15 days, the nodal explants were excised, and transformation was carried out as described [39] based on the protocol developed by Horsch et al. [40]. The *Agrobacterium tumefaciens* strain used for *A. oleracea* genetic transformation was LBA4404 [41], harboring Bin 19 plasmids [42] (GenBank Acc. N. U09365) containing the ORF 12 (*rolC* gene) from the wild type *A. rhizogenes* 1855 Ri plasmid [43] (Figure 8). The *A. tumefaciens* strain was grown on YEB medium (5 g/L Beef Extract, 1 g/L Yeast Extract, 5 g/L Peptone, 5 g/L Sucrose, 0.5 g/L MgSO_4_) supplemented with 100 μg/mL kanamycin and 40 μg/mL rifampicin. The shoots growing on regeneration selective medium LS1 (LS medium supplemented with 1 mg/L 6−benzyl aminopurine (BAP), 0.1 mg/L naphthaleneacetic acid (NAA) and 10 mg/L kanamycin) were isolated and individually transferred to De Wit culture tubes containing hormone-free selective medium.

Prior to the molecular analysis, putative transformants were checked for the persistence of *A. tumefaciens* in plant tissues by incubating 100 μL of leaf macerate in 3 mL of YEP medium (10 g/L Bacto Peptone, 10 g/L yeast extract, 5 g/L NaCl), with the addition of 20 mg/L rifampicin and 50 mg/L kanamycin, in agitation in the dark at 28 °C for 72–96 h [39].

Confirmed transgenics were micropropagated in Wavin containers (Lab Associates B.V.) on both selective and non−selective LS medium and maintained in a growth chamber at 24 ± 1 °C, with a 16 h light–8 h dark photoperiod.

For the analysis of secondary metabolites, 25 g each (fw) of aerial part and roots from both individual clones and controls were dried at 50 °C and stored in vacuum-−sealed plastic bags until use.

### 4.2. Molecular Analysis of Transformants

The presence of the *rolC* gene in regenerated plants was assessed by direct PCR (Phire Plant Direct PCR Kit, Thermo Fisher Scientific Inc., Waltham, MA, USA) with the following primers: *rolC* forward: 5′−ATGGCTGAAGACGACCTGTGT−3′ and *rolC* reverse: 5′−TCATCGAGAGTCACATCATGC−3′. Prior to the amplification of the transgene sequence, a control reaction with primers for a highly conserved 297 bp region of chloroplast DNA, provided in the kit, was performed, as recommended by the manufacturer.

For the analysis of *rolC* expression, total RNA was extracted from plant tissue with the GeneJet Plant RNA Purification kit (Thermo Fisher Scientific Inc., Waltham, MA, USA), genomic DNA was removed with the RapidOut DNA Removal kit (ThermoFisher Scientific Inc.) and RNA quantitation was carried out with the Qubit™ fluorometer (Qubit™ RNA BR Assay kit, Thermo Fisher Scientific Inc.). The absence of genomic DNA was verified by PCR on the RNA samples with primers for the chloroplast *psbA* gene (*psbA* forward: 5′−GAAAACCGTCTTTACATTGGA−3′ and *psbA* reverse: 5′−AGTTGTGAGCATTACGTTCAT−3′).

An amount of 1 μg of total RNA was reverse transcribed (QuantiTect Reverse Transcription kit, Qiagen GmbH, Hilden, Germany) in a final volume of 20 μL, and quantitative real−time PCR analysis was performed on 5 μL of a 1:5 dilution of the cDNA template with a custom TaqMan^®^ Gene Expression Assay (Thermo Fisher Scientific Inc.). The following primers and internal oligonucleotide probes were used: *rolC*−TaqMan forward 5′−TTCGGTTACGCGGATCCTAT−3′, *rolC*−TaqMan reverse 5′−CACGCCCAGGGAAAGAAAAT−3′, *rolC* probe 5′−CGGAGCGCCTACTTCGCTGCA−3′. Amplification was carried out in a final volume of 20 μL with a QuantStudio 7 Real-Time PCR System (Thermo Fisher Scientific Inc.).

### 4.3. Preparation of Food−Grade Extracts

The aerial parts and roots of both control (CTR) and transformed samples (C123, C127, C129, and C132) were cryo−pulverized using a blade mill in the presence of liquid nitrogen to preserve bioactive compounds and prevent enzymatic degradation. An amount of 200 mL of 96% ethanol/deionized water (80:20, *v*/*v*) mixture were added to one gram of each sample. Ultrasound−assisted extraction was performed in a heated water bath at 60 °C using a titanium probe sonicator (3 mm) set at a power of 200 W and 30% amplitude (Vibra Cell™ Sonics Materials, Inc., Danbury, CT, USA). Sonication lasted for 5 min to enhance the efficiency of compound release while minimizing thermal degradation. Following extraction, the samples were centrifuged at 3000× *g* for 10 min at 15 °C. The supernatant was collected and filtered through Whatman No. 1 filter paper directly into a rotary evaporator flask. The extraction process was repeated two additional times to maximize compound recovery. The combined supernatants were evaporated to dryness using a rotary evaporator. The resulting dry extracts were then placed in a vacuum desiccator overnight in the presence of anhydrous sodium sulfate. The next day, the extracts were weighed to determine the extraction yield and subsequently stored in amber-sealed vials with a nitrogen headspace to prevent oxidation and degradation before further analysis.

### 4.4. Phytochemical Analyses

#### 4.4.1. Total Phenolics

The total phenolic content was determined following the method described by Ingegneri et al. [44]. Ten microliters of each extract (1.25–10 mg/mL) were added to 50 μL of Folin–Ciocalteu reagent in a borosilicate test tube. After adding 45 μL of deionized water, the samples were vortexed and incubated for 3 min. Subsequently, 50 μL of 10% sodium carbonate was added, and the samples were incubated in the dark at room temperature (RT) for 1 h and vortexed every 10 min. The samples were then plated in a 96−well plate, and absorbance was recorded at 785 nm using a Multiskan™ GO Microplate Spectrophotometer (Thermo Scientific, Waltham, MA, USA). Gallic acid (75–600 µg/mL) was used as a reference standard, and results were expressed as GAE/100 g of dry extract (DE).

#### 4.4.2. Total Flavonoids

The flavonoid content of the test samples was determined according to Lenucci et al. [45] using rutin as the reference standard (125–1000 μg/mL). The test was carried out in borosilicate tubes, in which 450 μL of deionized water, 50 μL of the sample (1.25–10 mg/mL) and 30 μL of 5% sodium nitrite were added. The samples were then incubated for 5 min. After adding 60 μL 10% aluminum chloride, the samples were incubated again for an additional 6 min. Finally, 200 μL of 1 M sodium hydroxide and 210 μL of deionized water were added. After shaking the samples on the vortex, the absorbance was recorded at 510 nm using a UV–Vis spectrophotometer (UV–1601, Shimadzu, Kyoto, Japan). The flavonoid content was expressed as g RE/100 g DE.

#### 4.4.3. Vanillin Index

The vanillic index test, performed according to Smeriglio et al. [46], was used to determine the flavan−3−ols content in the samples. The extracts were diluted in 2 mL of 0.5 M sulfuric acid and loaded onto Sep−Pak C18 solid-phase extraction columns (Waters S.p.A, Milan, Italy), which had been previously activated with 2 mL of methanol and 2 mL of deionized water.

After washing with 5 mM sulfuric acid and removing any residual water by air insufflation, the samples were slowly eluted with 5 mL of methanol into a glass tube. Then, 6 mL of a 4% methanolic vanillin solution was added to 1 mL of the sample eluate, and the test tube was placed in a thermostatic bath at 20 °C for 10 min. Subsequently, 3 mL of concentrated hydrochloric acid was added, and the samples were incubated at room temperature for 15 min. Absorbance was measured at 500 nm using the same spectrophotometer described in Section 4.4.2. Results were expressed as g CatE/100 g DE by using a standard calibration curve (125–500 µg/mL).

#### 4.4.4. Proanthocyanidins

For the determination of proanthocyanidins [46], the same solid−phase extraction procedure described in Section 4.4.3 was performed. The samples were eluted with 3 mL of methanol directly into a 100 mL flask covered with aluminum foil containing 9.5 mL of absolute ethanol. Subsequently, 12.5 mL of ferrous sulfate heptahydrate solution (300 mg/L) dissolved in concentrated HCl was added, and the flask was allowed to boil at reflux for 50 min. After cooling, the absorbance of the test samples was measured at 550 nm using the same instrument mentioned in Section 4.4.2. The proanthocyanidin content was calculated by subtracting the absorbance of the samples treated under these conditions from that of the same samples treated identically but allowed to stand in ice (representing the basal anthocyanin content). Proanthocyanidin content was quantified using the molar extinction coefficient of cyanidin chloride (ε = 34,700 L·mol^−1^·cm^−1^). The results were expressed as g of cyanidin chloride equivalents (CyE)/100 g DE.

#### 4.4.5. LC−DAD−ESI−MS Analysis

Phytochemical profile characterization was performed using an Agilent 1200 Series system equipped with a binary pump, a thermostatic autosampler, a thermostatic column oven, a diode array detector, and an ion trap mass spectrometer (Agilent Technologies, Santa Clara, CA, USA). Chromatographic separation was carried out on a Luna Omega PS C18 column (150 mm × 2.1 mm, 5 μm; Phenomenex, Torrance, CA, USA) maintained at 40 °C, using 0.1% formic acid (A) and acetonitrile (B) as the mobile phase, following the elution program described by Nascimento et al. [47]: 0–5 min, 95% (A); 5–10 min, 63% (A); 10–12 min, 5% (A); 12–13 min, 95% (A); 13–15 min, 95% (A). The flow rate was set at 0.4 mL/min, and the injection volume was 2 μL.

The UV–Vis spectra of the analytes were recorded within a wavelength range of 190–600 nm. Chromatographic data were collected at 210, 260, 292, 330, 370, and 520 nm to ensure the detection of various phytochemical classes of interest.

Mass spectrometric analysis was performed using an ion trap mass spectrometer (model 6320, Agilent Technologies, Santa Clara, CA, USA) operating in full−scan mode (90–2000 m/z) under both positive and negative electrospray ionization (ESI) conditions. The following parameters were applied: capillary voltage 3.5 kV, atomization pressure (N_2_) 40 psi, dry gas temperature 350 °C, dry gas flow 9 L/min, and skimmer voltage 40 V. Data acquisition was carried out using Agilent ChemStation software version B.01.03 and Agilent Trap Control software version 6.2.

Tentative compound identification was achieved by comparing the retention times, UV–Vis spectra, and mass spectra of detected analytes with commercially available HPLC−grade standards (see footnotes of Table 3 and Table 4 for details), as well as with literature data and freely available online UV–Vis and mass spectra databases, including SpectraBase^®^, PhytoHub, ReSpect for Phytochemicals, MassBank, and PubChem.

### 4.5. Determination of Antioxidant and Anti−Inflammatory Activity

The antioxidant and anti−inflammatory activities of *A. oleracea* extracts were evaluated using a variety of colorimetric, turbidimetric, and enzymatic in vitro assays, each based on different environments and reaction mechanisms. Specifically, the tests, carried out according to Ingegneri et al. [44] and Cornara et al. [48], were categorized as follows: (i) Electron transfer−based reactions, such as the ferric−reducing antioxidant power (FRAP) assay; (ii) hydrogen atom transfer−based reactions, including oxygen radical absorption capacity (ORAC) and β−carotene bleaching (BCB) assays; (iii) mixed−mechanism reactions, involving both electron and hydrogen atom transfer, such as the Trolox equivalent antioxidant capacity (TEAC) and 1,1−Diphenyl−2−picrylhydrazyl (DPPH) assays.

Additionally, iron−chelating activity (ICA), given the crucial role of iron as a cofactor in many enzymatic oxidative reactions, was assessed according to Cornara et al. [48].

The anti−inflammatory activity was evaluated according to Smeriglio et al. [46] using the bovine serum albumin denaturation assay (ADA) and the protease inhibition assay (PIA).

Results were expressed as IC_50_ (µg/mL) for oxidative and/or inflammatory activity, with 95% C.L. calculated using the Litchfield and Wilcoxon test via PHARM/PCS software (version 4; Consulting, Wynnewood, PA, USA). The concentration ranges reported for each test represent the final concentrations of the samples and reference standards in the reaction mixture. Deionized water, used to dilute the samples, was used as blank. Unless otherwise specified, the reported concentration ranges apply to extracts from both the aerial parts and the roots.

#### 4.5.1. FRAP

Five microliters of the sample (60–480 μg/mL) were added to 200 μL of FRAP reagent, which consisted of a preheated (37 °C) mixture of 10 mM 2,4,6−Tris(2−pyridyl) −s−triazine dissolved in 40 mM HCl, 20 mM FeCl_3_·6H_2_O, and 300 mM acetate buffer (pH 3.6) 1:1:10 (*v*/*v*/*v*). After 4 min of incubation in the dark at RT, the absorbance was recorded at 593 nm using the same instrument described in Section 4.4.1. Trolox was used as the reference standard (1.25–10 μg/mL).

#### 4.5.2. DPPH

Briefly, 3.75 μL of the sample (30–240 μg/mL) was added to 150 μL of a methanolic DPPH solution (10^−4^ M). After mixing, the reaction mixture was incubated in the dark at RT for 20 min. Absorbance was measured at 517 nm using the same instrument described in Section 4.4.1. Trolox was used as the reference standard (2.5–20 μg/mL).

#### 4.5.3. TEAC

The blue−green-colored radical reagent was obtained by mixing 1.7 mM ABTS^•+^ radical solution with 4.3 mM ammonium persulfate 1:5 (*v*/*v*), followed by incubation in the dark at RT for 12 h. Before use, the solution was diluted to achieve an absorbance of 0.7 ± 0.02 at 734 nm and used within four hours. An amount of 10 μL of the sample (60–480 μg/mL) was added to 200 μL of the radical solution. After 6 min, the absorbance was recorded at 734 nm using the same instrument reported in Section 4.4.1. Trolox was used as the reference standard (1.25–10 μg/mL).

#### 4.5.4. ORAC

An amount of 20 µL of the sample (1.56–12.5 µg/mL), diluted in PBS, was added to 120 µL of a freshly prepared 117 nM fluorescein solution in PBS. After an initial incubation at 37 °C for 15 min, 60 µL of a freshly prepared 40 mM 2,2′−Azobis(2−methylpropionamidine) dihydrochloride solution, also dissolved in PBS, was rapidly added. Fluorescence was measured every 30 s for 90 min (λ_ex_ 485 nm; λ_em_ 520 nm) using a Varioskan^TM^ LUX multimode microplate reader (Thermo Fischer Scientific Inc.). Trolox was used as the reference standard (0.25–2 µg/mL).

#### 4.5.5. BCB

Two emulsions were prepared, both containing linoleic acid and Tween 40; one emulsion included β−carotene, while the other did not, serving as a negative control (white emulsion). The reaction was carried out by mixing 5 mL of the β−carotene−containing emulsion with 200 μL of the sample (12.5–100 μg/mL), reference standard (BHT, 0.063–0.5 μg/mL), or blank in borosilicated glass tubes. The reaction mixture was then placed in a water bath at 50 °C under continuous stirring. Absorbance was recorded at the initial time point (T_0_) and every 20 min for a total of 2 h using the plate reader described in Section 4.4.1.

#### 4.5.6. ICA

Fifty microliters of the sample (175–1400 μg/mL) was added to 25 μL of a 2 mM FeCl_2_·4H_2_O solution. The mixture was incubated at room temperature for 5 min. Then, 50 μL of a 5 mM ferrozine solution and 1.375 mL of deionized water were added. The samples were vortexed and incubated for an additional 10 min at RT. Absorbance was measured at 562 nm using the plate reader described in Section 4.4.1. Ethylenediaminetetraacetic acid (EDTA, 1.5–12.5 μg/mL) was used as the reference standard.

#### 4.5.7. ADA

Four hundred and eighty microliters of the sample (1–8 mg/mL for aerial parts and 0.125–2 mg/mL for roots) were added to 600 µL of a 0.4% BSA solution dissolved in PBS (pH 5.3). An initial spectrophotometric reading (T_0_) was taken at 595 nm using the plate reader described in Section 4.5.4. The samples were then incubated in a water bath under agitation at 70 °C for 30 min to induce the protein denaturation process.

Diclofenac sodium (7.5–60 µg/mL) was used as the reference standard, while PBS served as a negative control. At the end of the reaction, absorbance was measured again, and the percentage of protein denaturation inhibition was calculated.

#### 4.5.8. PIA

An amount of 200 µL of the sample (62.5–1000 µg/mL) was mixed with 12 µL of trypsin (10 µg/mL) and 188 µL of Tris−HCl buffer (25 mM, pH 7.5) and incubated in a water bath for 5 min. Then, 200 µL of 0.8% casein was added, and the reaction mixture was further incubated at 37 °C in the water bath for 20 min. After the incubation, 400 µL of perchloric acid was added to stop the reaction and precipitate the enzyme. The mixture was then centrifuged at 3500× *g* for 10 min, and the absorbance of the supernatant was measured at 280 nm against a blank consisting of deionized water alone, using the instrument described in Section 4.4.2. Diclofenac sodium (10–80 µg/mL) was used as the reference standard.

### 4.6. Statistical Analyses

Data are presented as the mean ± standard deviation from three independent experiments performed in triplicate (*n* = 3). Statistical analysis was conducted using a one-way analysis of variance (ANOVA), followed by the Student–Newman–Keuls post-hoc test to determine significant differences. A *p*-value ≤ 0.05 was considered statistically significant.

Data processing was performed using SigmaPlot 12.0 software (Systat Software Inc., San Jose, CA, USA). Chemometric analyses, including dendrogram generation and hierarchical clustering analysis (HCA), were carried out using JMP software (version 17, SAS Institute Inc., Cary, NC, USA). To evaluate differences in phytochemical profiles, the Euclidean distance was calculated, and hierarchical clustering analysis was carried out using Ward’s variance-minimization method.

## 5. Conclusions

This study provides novel insights into the role of the *A. rhizogenes rolC* gene in modulating the secondary metabolite profile and biological activities of *A. oleracea*, with a specific focus on food−grade extracts derived from aerial parts and roots.

The transformation significantly influenced the phytochemical composition in a plant part−dependent manner. Aerial part extracts, rich in flavonoids and spilanthol, exhibited moderate antioxidant and anti−inflammatory activities, with a particularly strong iron−chelating capacity attributed to the high flavonoid content. Root extracts, although characterized by a lower diversity of polyphenolic compounds, demonstrated superior radical scavenging and anti−inflammatory activities, likely related to the presence of specific alkylamides and residual flavonoids.

These findings highlight that distinct plant tissues can offer differentiated functional properties, which can be selectively exploited depending on the desired application. Aerial part extracts may represent promising candidates for antioxidant nutraceuticals and functional foods, while root extracts could be particularly valuable in the development of anti-inflammatory formulations.

The adoption of food−grade extraction processes strengthens the potential translational application of these extracts, ensuring their safety for human consumption.

Further investigations are warranted to standardize production processes, to fully characterize the biosynthetic pathways and regulatory networks involved, to validate the observed effects in whole plants and under field conditions, and to explore the in vivo biological activities of these extracts, thereby paving the way for the development of innovative health-promoting products based on *A. oleracea*.

## Figures and Tables

**Table 1:** Quantification of *rolC* mRNA in transgenic *A. oleracea* clones as determined by real−time PCR.

Clone	fg *rolC* mRNA/μg Total RNA
*rolC*−123	1.6
*rolC*−127	9.14
*rolC*−129	0.105
*rolC*−132	0.282

**Table 2:** Phytochemical screening of hydroalcoholic extracts from the aerial parts and roots of *A. oleracea*, including transgenic (C123, C127, C129, and C132) and non−transgenic (CTR) specimens. The results are expressed as the mean ± standard deviation from three independent experiments performed in triplicate (*n* = 3).

Extracts	Total Phenols (g GAE ^a^/100 g DE ^b^)	Total Flavonoids (g RE ^c^/100 g DE)	Vanillic Index (g CE ^d^/100 g DE)	Proanthocyanidins (g CcE ^e^/100 g DE)	PI ^f^
*Aerial parts*					
CTR	4.22 ± 0.34	2.01 ± 0.14	1.61 ± 0.03	0.01 ± 0.00	161.00
C123	4.33 ± 0.13	2.97 ± 0.22 **	2.66 ± 0.03 ***	0.02 ± 0.00 **	133.00
C127	2.62 ± 0.15 ***	2.46 ± 0.02 **	1.84 ± 0.02 *	0.02 ± 0.00 **	92.00
C129	2.79 ± 0.08 ***	2.24 ± 0.01	1.34 ± 0.03 **	0.03 ± 0.00 ***	44.67
C132	4.48 ± 0.26	2.22 ± 0.03	1.66 ± 0.02	0.01 ± 0.00	166.00
*Roots*					
CTR	8.44 ± 0.12	2.25 ± 0.13	0.318 ± 0.01	0.02 ± 0.00	15.90
C123	4.27 ± 0.18 ***	0.85 ± 0.01 ***	0.068 ± 0.00 ***	0.01 ± 0.00 **	6.80
C127	3.25 ± 0.03 ***	1.66 ± 0.04 ***	0.773 ± 0.02 ***	0.01 ± 0.00 **	77.30
C129	3.05 ± 0.17 ***	0.73 ± 0.06 ***	0.455 ± 0.02 ***	0.01 ± 0.00 **	45.50
C132	2.86 ± 0.11 ***	0.74 ± 0.02 ***	0.273 ± 0.01 **	0.01 ± 0.00 **	27.30

^a^ GAE—gallic acid equivalents; ^b^ DE—dry extract; ^c^ RE—rutin equivalents; ^d^ CE—catechin equivalents; ^e^ CcE—cyanidin chloride equivalents; ^f^ PI—Polymerization Index = Vanillin Index/Proanthocyanidins; * *p* < 0.05 vs. CTR; ** *p* < 0.01 vs. CTR; *** *p* < 0.001 vs. CTR.

**Table 3:** LC−DAD−ESI−MS analysis of hydroalcoholic extracts from the aerial parts of transgenic (C123, C127, C129, and C132) and non−transgenic (CTR) *A. oleracea* plants. Analyses were performed in triplicate (*n* = 3).

Metabolite	RT ^a^ min	[M-H]^−^ m/z	[M-H]^+^ *m*/*z*	CTR	C123	C127	C129	C132
Caffeoylmalic acid	1.0	−	297	+	−	−	+	+
Neochlorogenic acid ^b^	1.6	353	−	−	+	−	−	−
Cyanidin 3−*O*−diglucoside−5−o−glucoside	21.3	−	774	+	−	+	+	+
Tryptophan	22.6	−	205	−	+	−	+	+
Quercetin−3−*O*−(diacetyl−glucopyranoside)	24.1	547	−	−	−	−	+	+
Cyanidin−3−*O*−glucoside ^b^	25.0	−	450	−	+	+	+	+
3,5−di−*O*−caffeoylquinic acid	26.6	515	−	−	+	−	−	−
Quercetin−3−*O*−glucuronide (Miquelianin)	29.0	−	479	+	−	−	−	−
Delphinidin−3−*O*−glucuronide	30.0	−	508	−	−	+	+	+
Quercetin−3−*O*−(3−*O*−acetyl−β−D−glucopyranoside) isomer 1	30.3	−	521	+	+	−	−	−
Quercetin−rutinoside (Rutin) ^b^	31.2	−	611	+	+	−	−	−
Quercetin−*O*−acetil−glucopyranosyl−*O*−rhamnopyranoside	32.1	−	667	+	−	−	−	−
Quercetin 3−*O*−(6″−malonil−glucoside) 7−*O*−glucoside	32.4	−	713	+	−	−	−	−
Quercetin 3−sambubioside	32.8	651	653	−	−	+	−	−
Feruloyl malic acid	33.2	309	311	−	−	+	+	+
Quercetin−3−*O*−(3−*O*−acetil−β−D−glucopyranoside) isomer 2	33.8	519	−	−	+	+	+	+
4,5−di−*O*−caffeokyninic acid	34.7	−	517	−	−	+	−	+
Quercetin−acetyl esoside	37.0	505	−	−	+	+	+	+
cis−Clovamide	38.5	358	−	+	+	+	+	+
Quercetin−*O*−(*O*−acetyl−glucopyranosyl rhamnopyranoside)	42.5	−	653	−	−	+	+	−
(+)−Catechin 3−*O*−glucoside	43.6	−	453	+	−	+	+	+
Quercetin 3′−*O*−sulfate	46.1	381	−	+	+	−	−	+
Methyl ester of *O*−caffeoyl−*O*−sinapoilkinic acid	49.6	573	−	−	+	−	+	+
(E)−*N*−isobutylundeca−2−(E)−en−8,10−dynamide	50.2	−	232	+	+	−	−	−
Dihydrospilanthol	51.7	−	224	+	−	−	+	−
Spilanthol ^b^	52.9	−	222	+	+	+	+	+
(2E,4E)−*N*−isobutylundeca−2,5−dien−8,10−diynamide	53.5	−	230	−	−	−	+	−
(2E,7Z)−*N*−Isobutyl−2,7−tridecadiene−10,12−diynamide	54.4	−	258	+	+	+	+	+
(7Z)−*N*−(isobutyl)−7−tridecene−10,12−dynamide	54.9	−	260	+	−	−	−	−
*N*−isobutyl deca−2,8−dien−6−inamide	55.3	−	220	+	+	+	+	+
Quercetin−3−O−deoxyhexosyl(1−2)deoxyhexoside	57.9	593	−	+	−	−	−	−
Dodeca−2,4,8,10−tetraenoic isobutylamide acid	59.3	−	248	+	+	+	+	+
((2E)−*N*−(2−Methylbutyl)−2−undecene−8,10−diynamide	63.2	−	246	+	−	−	+	−
(2E,6E,8E)−*N*−(2−methylbutyl)deca−2,6,8−trienamide	66.4	−	236	+	+	−	+	−
*N*−phenylethyl−2,3−epoxy−6,8−nonadiynamide	68.8	−	508	−	−	+	+	+
*N*−phenylethyl−2,3−epoxy−6,8−nonadiynamide	76.2	−	268	+	+	−	−	+
Quercetin−*O*−(glucopyranosyl−*O*−acetylglucopyranoside)	79.3	−	669	−	+	−	+	+

^a^ RT—Retention time; +—detected; −—not detected; ^b^ Absolute identification was performed using reference standards purchased from Merck (Darmstadt, Germany) or Extrasynthase (Genay, France).

**Table 4:** LC−DAD−ESI−MS analysis of hydroalcoholic extracts from the roots of transgenic (C123, C127, C129, and C132) and non−transgenic (CTR) *A. oleracea* plants. Analyses were performed in triplicate (*n* = 3).

Metabolite	RT ^a^ min	[M−H]^−^ *m*/*z*	[M−H]^+^ *m*/*z*	CTR	C123	C127	C129	C132
Caffeoylmalic acid	1.0	−	297	+	+	+	+	+
Quercetin−3−*O*−glucoside ^b^	23.3	462	−	+	+	+	+	+
Quercetin−3−*O*−(3−*O*−acetyl−β−D−glucopyranoside) isomer 1	30.3	−	521	+	+	+	+	+
Quercetin−rutinoside (Rutin) ^b^	31.2	−	611	+	+	−	−	+
Quercetin−*O*−acetil−glucopyranosyl−*O*−rhamnopyranoside	32.1	−	667	+	+	−	−	+
Quercetin−*O*−(*O*−acetyl− glucopyranosyl rhamnopyranoside)	32.8	651	653	+	+	−	−	−
Quercetin−3−*O*−(3−*O*−acetil−β−D− glucopyranoside) isomer 2	33.8	519	−	+	+	+	+	+
4,5−di−*O*−caffeokyninic acid	34.7	−	517	+	+	−	−	−
cis−Clovamide	38.5	358	−	+	+	+	+	+
(E)−*N*−isobutylundeca−2−(E)−en−8,10−dynamide	50.2	−	232	+	+	+	+	+
Dihydrospilanthol	51.7	−	224	+	−	+	−	−
Spilanthol ^b^	52.9	−	222	+	+	+	+	+
2E,4E)−*N*−isobutylundeca−2,5−dien−8,10−diynamide	53.5	−	230	+	−	+	−	−
(2E,7Z)−*N*−Isobutyl−2,7−tridecadiene−10,12−diynamide	54.4	−	258	+	+	+	+	+
*N*−isobutyl deca−2,8−dien−6−inamide	55.3	−	220	+	+	+	+	+
Dodeca−2,4,8,10−tetraenoic isobutylamide acid	59.3	−	248	+	+	+	+	+

^a^ RT—Retention time; +—detected; −—not detected; ^b^ Absolute identification made with the reference standard purchased from Merck (Darmstadt, Germany) or Extrasynthase (Geney, France).

**Table 5:** Quantitative analysis of spilanthol in hydroalcoholic extracts from aerial parts and roots of transgenic (C123, C127, C129, and C132) and non−transgenic (CTR) *A. oleracea* plants by HPLC−DAD. Results are expressed as mean ± standard deviation from three independent experiments, each performed in triplicate (*n* = 3).

Hydroalcoholic Extracts	Aerial Parts (g/100 g)	Roots (g/100 g)
CTR	5.70 ± 0.08	0.077 ± 0.00
C123	2.90 ± 0.04 ***	0.064 ± 0.00 **
C127	1.29 ± 0.02 ***	0.019 ± 0.00 ***
C129	0.17 ± 0.01 ***	0.051 ± 0.00 ***
C132	4.88 ± 0.05 **	0.028 ± 0.00 ***

** *p* < 0.01 vs. CTR; *** *p* < 0.001 vs. CTR.

**Table 6:** Antioxidant properties of hydroalcoholic extracts of aerial parts and roots of transgenic (C123, C127, C129, and C132) and non−transgenic (CTR) *A. oleracea* plants. IC_50_ values (and 95% confidence intervals) are shown. Statistical comparisons were performed between each transgenic line and the non−transgenic control within the same plant part (AP or R). No direct comparisons were made between aerial parts and roots. Asterisks indicate significant differences versus the corresponding control: *** *p* < 0.001.

Extracts	DPPH	FRAP	ICA	TEAC	ORAC	BCB
*Aerial parts*	IC_50_ mg/mL				IC_50_ µg/mL	
CTR	0.44 (0.32–0.59)	0.19 (0.16–0.23)	0.18 (0.15–0.22)	1.44 (0.91–2.29)	8.04 (2.50–14.40)	35.65 (29.92–42.47)
C123	0.94 (0.57–1.56)	0.20 (0.08–0.47)	0.30 (0.24–0.38)	1.50 (0.91–2.49)	7.90 (1.02–11.25)	24.73 (17.18–35.61)
C127	1.05 (0.61–1.79)	0.35 (0.29–0.41)	0.15 (0.13–0.18)	1.14 (0.77–1.69)	7.90 (0.48–13.01)	47.82 (34.25–66.77)
C129	0.59 (0.41–0.86)	0.26 (0.22–0.32)	0.33 (0.26–0.41)	1.30 (0.84–2.00)	10.73 (9.15–12.58)	60.33 (40.25–90.44)
C132	0.60 (0.40–0.88)	0.20 (0.16–0.24)	0.24 (0.19–0.29)	0.56 (0.41–0.77)	6.74 (6.60–8.12)	107.77 (65.06–178.52)
*Roots*	IC_50_ (mg/mL)				IC_50_ µg/mL	
CTR	0.25 (0.19–0.33)	0.08 (0.07–0.09) ***	0.31 (0.25–0.39) ***	0.23 (0.16–0.34) ***	3.87 (2.30–6.50)	13.39 (10.38–17.26) ***
C123	0.89 (0.55–1.45)	0.09 (0.06–0.12)	0.74 (0.57–0.97) ***	0.33 (0.26–0.43) ***	9.90 (7.66–12.79)	15.89 (12.51–20.19) ***
C127	0.44 (0.30–0.66)	0.14 (0.12–0.17) ***	0.71 (0.54–0.92) ***	0.37 (0.28–0.48) ***	11.58 (8.49–15.80)	22.38 (16.30–31.08) ***
C129	0.34 (0.26–0.46)	0.15 (0.12–0.17) ***	0.74 (0.58–0.95) ***	0.44 (0.33–0.59) ***	11.51 (8.69–15.25)	37.89 (25.50–56.31)
C132	0.57 (0.38–0.86)	0.14 (0.12–0.16)	1.38 (1.06–1.79) ***	0.39 (0.30–0.51) ***	10.02 (7.34–13.68)	20.42 (14.13–29.50) ***
	IC_50_ (µg/mL)					
**Standard ^a^**	6.09 (5.71–8.18)	3.74 (1.55–5.01)	6.54 (5.74–7.46)	3.95 (2.39–6.09)	0.79 (0.13–1.68)	0.37 (0.15–0.76)

^a^ Trolox for DPPH, FRAP, TEAC, and ORAC assay; EDTA for iron-chelating activity (ICA); and BHT for β-carotene bleaching (BCB) assay.

**Table 7:** Anti−inflammatory properties of hydroalcoholic extracts from aerial parts and roots of transgenic (C123, C127, C129, and C132) and non−transgenic (CTR) *A. oleracea* plants. The IC_50_ values (and 95% confidence intervals) are shown. Statistical comparisons were performed between each transgenic line and the non−transgenic control within the same plant part (AP or R). No direct comparisons were made between aerial parts and roots. Asterisks indicate significant differences versus the corresponding control: *** *p* < 0.001.

	ADA	PIA
*Aerial parts*	IC_50_ mg/mL	
CTR	3.56 (2.90–4.36)	0.38 (0.31–0.46)
C123	2.92 (2.28–3.75)	0.48 (0.36–0.56)
C127	3.94 (2.99–5.20)	0.51 (0.39–0.66)
C129	4.91 (3.70–6.53)	0.46 (0.38–0.55)
C132	7.24 (5.78–9.08)	0.44 (0.35–0.54)
*Roots*	IC_50_ mg/mL	
CTR	2.05 (1.62–2.59) ***	0.18 (0.07–0.46)
C123	2.18 (1.69–2.82)	0.30 (0.26–0.36)
C127	0.72 (0.54–0.95) ***	0.53 (0.40–0.69)
C129	0.60 (0.43–0.83) ***	0.56 (0.43–0.74)
C132	1.14 (0.89–1.45) ***	0.37 (0.15–0.89)
Diclofenac sodium (IC_50_ µg/mL)	39.44 (25.28–54.21)	35.35 (21.95–56.93)

## Data Availability

The original contributions presented in this study are included in the article. Further inquiries can be directed to the corresponding authors.

## Abbreviations

The following abbreviations are used in this manuscript:

ADA	Albumin Denaturation Assay
AP	Aerial Parts
BCB	β−Carotene Bleaching Assay
BHT	Butylated HydroxyToluene
CcE	Cyanidin Chloride Equivalents
CE	Catechin Equivalents
CTR	Control (non-transformed plants)
DE	Dry Extract
DPPH	2,2−Diphenyl−1−picrylhydrazyl
EDTA	Ethylenediaminetetraacetic Acid
FRAP	Ferric Reducing Antioxidant Power
GAE	Gallic Acid Equivalents
HPLC-DAD	High−Performance Liquid Chromatography with Diode Array Detection
ICA	Iron−Chelating Activity
IC_50_	Half−maximal Inhibitory Concentration
LC-DAD-ESI-MS	Liquid Chromatography−Diode Array Detection−Electrospray Ionization−Mass Spectrometry
ORAC	Oxygen Radical Absorbance Capacity
PBS	Phosphate Buffered Saline
PCR	Polymerase Chain Reaction
PI	Polymerization Index
PIA	Protease Inhibition Assay
RE	Rutin Equivalents
RNA	Ribonucleic Acid
R	Roots
*rolC*	Root oncogenic locus C gene
RT	Retention Time
TEAC	Trolox Equivalent Antioxidant Capacity
